# Lip Volumization With Hyaluronic Acid: Comparative Ultrasonographic Evaluation of Cannula and Needle Techniques in a Multicenter Study

**DOI:** 10.7759/cureus.79325

**Published:** 2025-02-19

**Authors:** Claudia Gonzalez, Ester Callejas, Cristina Nuñez, Valeria Duque-Clavijo, Javier Murillo, Ernesto Barbosa, Sandra Suárez, Alejandro Coello

**Affiliations:** 1 Radiology, Highly Specialized Ultrasound Center, Bogota, COL; 2 Aesthetic Medicine, CM Clinic, Palma, ESP; 3 Aesthetic Medicine, Clínica Aureo, Palma, ESP; 4 College of Medicine, Universidad de los Andes, Bogota, COL; 5 Plastic Surgery, Barma Functional and Aesthetic Medicine, Bogota, COL; 6 Aesthetic Medicine, Grupo Presenza, Bogota, COL; 7 Facial and Body Aesthetics, Xtetic Clinique by Dr. Alejandro Coello, Mexico City, MEX

**Keywords:** cannula technique, hyaluronic acid, lip volumization, needle technique, patient satisfaction, ultrasound evaluation

## Abstract

Background: Lip volumization using hyaluronic acid (HA) is among the most popular aesthetic procedures due to the role of the lips in modern beauty standards. This study explored the ultrasound characteristics of HA deposits following injection using either cannula or needle techniques.

Methods: A multicenter, descriptive study involving 27 patients from five aesthetic centers in Colombia, Spain, and Mexico was conducted. Ultrasound evaluations were performed pre- and post-procedure to assess anatomical changes, HA deposit morphology, and vascular mapping with Doppler. Patients were randomly assigned to either the cannula technique (11 patients, representing 41% of the cohort) or the needle technique (16 patients, representing 59% of the cohort). Satisfaction was measured using a subjective scale (1-5).

Results: HA doses ranged from 0.4 to 1 cc. Among the 27 patients, 25 had a single type of deposit, while two patients presented with two different types simultaneously. Ultrasound examination revealed that 81% (22 patients) of labial arteries were located in the wet submucosa, 15% (four patients) in the dry submucosa, and 4% (one patient) intramuscularly. A total of nine patients presented with lip asymmetry, and two patients had lip hypotrophy; all 11 patients achieved correction, defined as the restoration of symmetrical lip volume and contour. Patient satisfaction was universally high, with 74% (20 patients) reporting complete satisfaction.

Conclusion: Ultrasound evaluation is an effective and safe method for assessing patients undergoing HA injections. Our observations indicate that injection techniques, whether using a needle or a cannula, influence the shape and location of HA deposits, with elongated deposits being more common with cannula use and round deposits predominantly associated with needle injections. Pre-procedure ultrasound allows for the identification of each patient’s specific lip anatomy, aiding in injection planning, while post-procedure ultrasound helps verify the location of deposits and detect potential complications. These findings highlight the importance of careful technique selection and thorough anatomical assessment to optimize safety and aesthetic outcomes. Future research should focus on larger samples and long-term follow-up to validate these observations and further enhance procedural safety in aesthetic medicine.

## Introduction

Lip volumization is currently one of the most popular aesthetic procedures, driven by the significance of the lips in the modern concept of beauty [1]. This procedure aims not only to restore lost volume but also to improve skin quality, fill perioral expression lines, and enhance lip luminosity and elasticity [2]. Although various hyaluronic acid (HA) injection techniques exist, the choice between using a cannula or a needle can significantly influence both the aesthetic outcomes and the safety of the procedure. However, patient preferences and aesthetic motivations, alongside the specialist's expertise, remain key factors in determining the approach and technique employed [3,4].

Advances in imaging technology, such as high-resolution ultrasound, have allowed for greater precision in understanding lip anatomy and optimizing HA application [5,6]. Ultrasound facilitates the precise localization of labial arteries, aids in evaluating filler distribution, and enables the early identification of complications [7].

The primary objective of this study is to describe the distribution and ultrasonographic appearance of HA following lip volumization procedures and to analyze how this appearance varies depending on the injection technique (cannula vs. needle). Secondary objectives include evaluating anatomical changes before and after the procedure using grayscale ultrasound, identifying the main aesthetic indications of patients seeking lip volumization, mapping and describing the course of labial arteries with color Doppler, analyzing morphological changes in the lips post-procedure, and assessing patient satisfaction levels.

By addressing these objectives, this study provides a comprehensive clinical and anatomical perspective on lip volumization. The findings emphasize the importance of tailoring injection techniques to individual patient needs, optimizing aesthetic outcomes, and ensuring the highest safety standards in aesthetic medicine [8].

## Materials and methods

Study design and participants

This was a multicenter descriptive study conducted at five aesthetic medicine centers: two in Bogotá, Colombia (Centro de Ultrasonido Altamente Especializado and Barma Medicina Funcional y Estética); two in the Balearic Islands, Spain (Clinica Aureo and CM Clinic); and one in Mexico City, Mexico (Xtetic Clinique by Dr. Alejandro Coello). Data collection was carried out over eight months, from February 1, 2024, to September 1, 2024. A total of 27 voluntary patients seeking lip volumization procedures for aesthetic reasons were recruited.

Inclusion and exclusion criteria

Adults of both sexes, aged 20 to 70 years, interested in a lip volumization procedure, were included. Patients with medical histories contraindicating the use of HA, pregnant or breastfeeding women, individuals with a history of allergy or hypersensitivity to HA or lidocaine, and those with infections or skin lesions on or near the lips were excluded.

Training and expertise of professionals

The injection procedures were performed by five physicians specializing in aesthetic medicine and one plastic surgeon, all with over 11 years of experience in injectable procedures. Ultrasound studies were conducted by two physicians specialized in aesthetic medicine trained in dermatologic ultrasound and a radiologist with over 13 years of experience in dermatologic ultrasound. The images obtained were reviewed, validated, and, if necessary, reinterpreted by the radiologist to ensure diagnostic accuracy.

Dermatologic ultrasound evaluation

High-resolution dermatologic ultrasound is the ideal diagnostic tool for a detailed evaluation of lip anatomy. All studies were conducted using an 18 MHz high-resolution linear transducer, following guidelines proposed by prior studies [9,10]. Grayscale, Doppler, and color Duplex ultrasound evaluations were performed both before and after the procedure.

Injection procedure

Patients were randomly assigned to one of two injection techniques: cannula or needle.

Cannula

**Figure 1:**
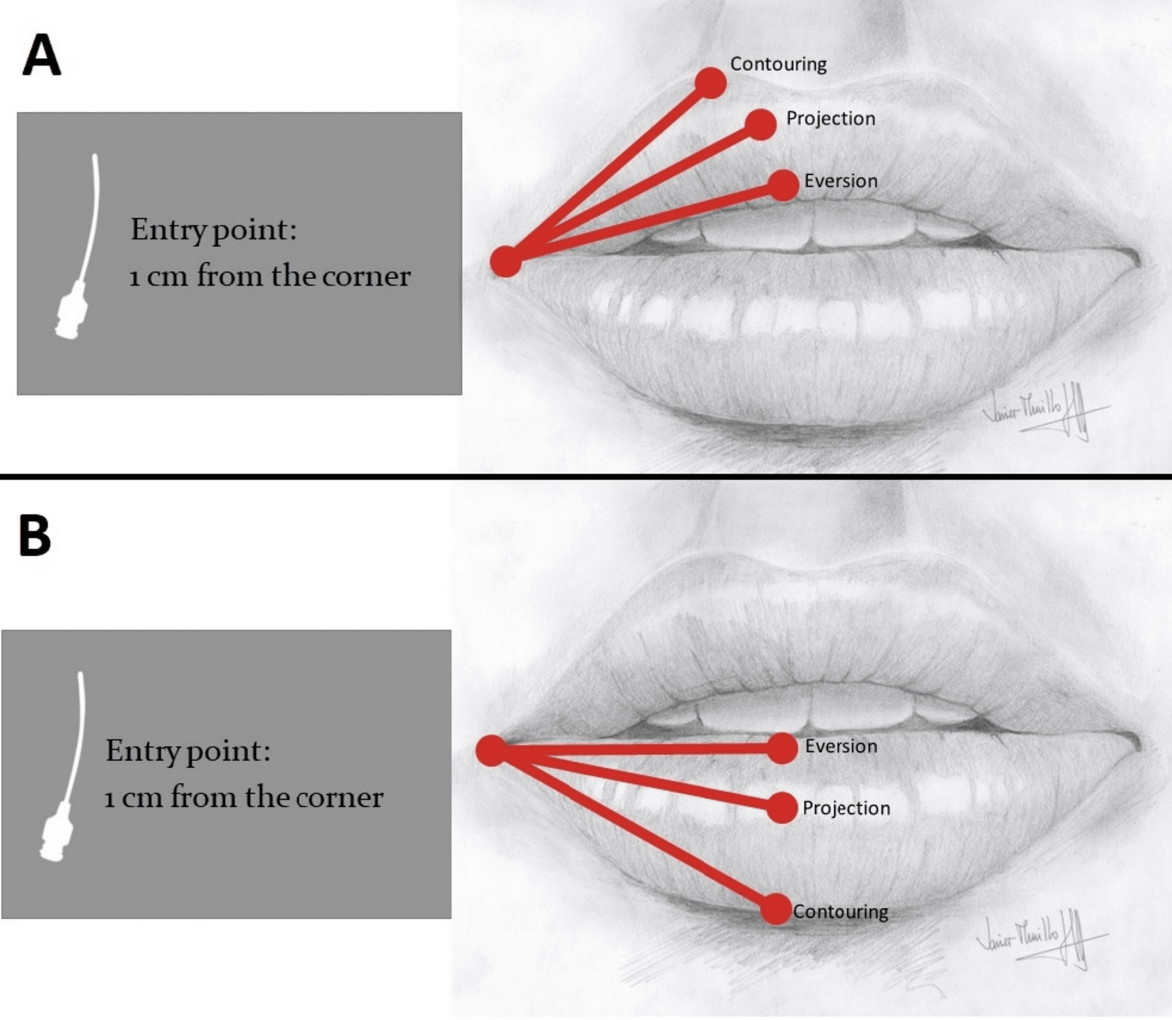
Diagram illustrating entry points and the volumization approach using the cannula technique A. Diagram illustrating entry points and the volumization technique for the upper lip using the cannula method. B. Diagram illustrating entry points and the volumization technique for the lower lip using the cannula method. Image Credits: Javier Murillo

In 11 patients (41% of the cohort), a 25 G x 50 mm cannula was used and a single entry point per hemilip at the commissure. Retrograde tracing was performed from the central zone toward the commissure, creating vectors in the appropriate plane and avoiding direct injections into the commissures (Figure 1).

Needle

**Figure 2:**
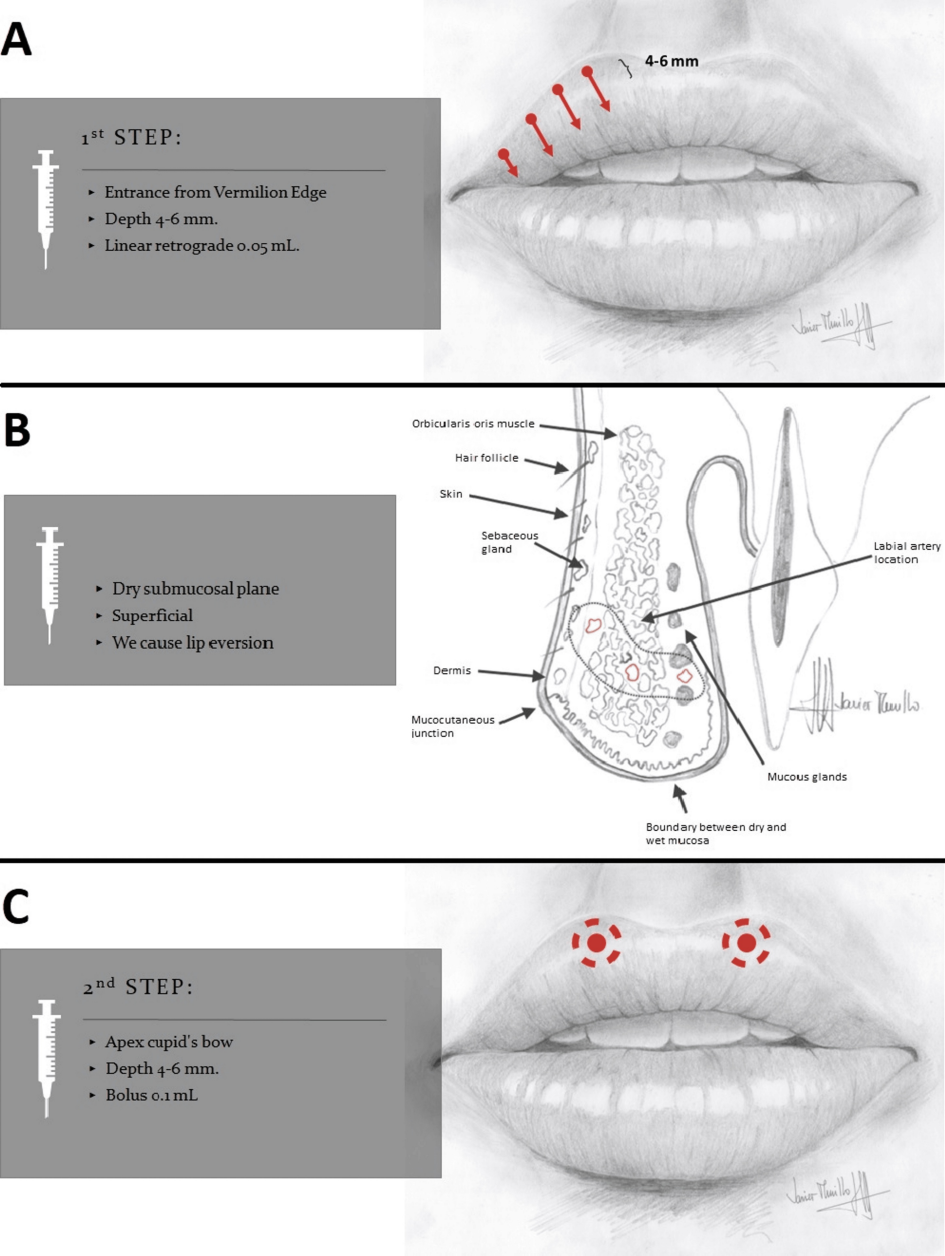
Diagram illustrating entry points and the volumization approach using the needle technique A. Diagram illustrating the entry points and the initial step of the volumization technique for the upper lip using the needle method. B. Diagram illustrating the anatomy of the plane in the upper lip being injected. C. Diagram illustrating the entry points and the second step of the volumization technique for the upper lip using the needle method. Image Credits: Javier Murillo

In 16 patients (59%), a 30 G x 13 mm needle was used, entering the vermilion border. The needle was inserted 4 mm deep, performing retrograde injections with 0.05 cubic centimeter (cc) volumes in the superficial submucosal plane to promote lip eversion. In addition, two 0.1 cc boluses were applied to the apex of the Cupid's bow at the same depth (Figure 2).

In all patients, the selection of the cannula or needle technique followed a standardized protocol:

Pre-procedure Ultrasound Evaluation

A grayscale ultrasound assessment of the lips was performed to rule out the presence of previous fillers, identify the different anatomical layers, and ensure clear visualization of the orbicularis oris muscle. A dynamic evaluation of the muscle was conducted to determine whether corrective myomodulatory effects were needed.

Vascular Mapping

A color Doppler duplex ultrasound analysis was performed before product injection to identify the location and course of the arteries.

Technique Selection

The choice between cannula and needle was based on the injector’s expertise, the patient's aesthetic goals, and the desired effect.

Injection Considerations Based on the Artery Depth

If the arteries were located deep within the wet mucosa, either technique could be used, ensuring that the injection was always superficial to the previously identified arterial course.

If the artery was superficial (in the dry mucosa), injections were performed with a short needle using a very superficial approach, following the recommendations of Lee et al. [11], while avoiding the course of the artery as identified via ultrasound.

Post-procedure Assessment

After the injection, an ultrasound evaluation in grayscale was performed to verify the even distribution of the product, and a Doppler analysis was conducted to confirm satisfactory blood flow.

This systematic approach aimed to optimize both safety and aesthetic outcomes by integrating ultrasound guidance for enhanced precision. All patients were treated with Restylane Kysse, a HA specifically designed for lips, using a standard volume of 0.4 to 1 cc per patient.

Pre- and post-procedure evaluation

A detailed physical examination was conducted prior to the procedure to document asymmetries, atrophy, lip inversion, perioral lines, barcode development, and any anatomical alterations.

In addition, pre- and post-procedure ultrasonographic studies were performed using an 18 MHz high-resolution linear transducer. Grayscale evaluations included assessments of atrophy, volume loss, asymmetry, dynamic barcode exploration, and previous filler deposits. In the color Doppler evaluation, the localization of labial arteries was identified. After HA application, a grayscale analysis described the location plane, filler morphology, changes in lip morphology, and absence of complications, particularly vascular occlusion, in the Doppler evaluation.

Patient satisfaction assessment

Patient satisfaction was assessed using a custom satisfaction scale developed specifically for this study. The scale ranged from 1 to 5, where 1 indicated "total dissatisfaction," 2 "dissatisfaction," 3 "moderate satisfaction," 4 "satisfaction," and 5 "complete satisfaction." Survey results were analyzed to determine the overall perception of the procedure.

Statistical analysis

Data were analyzed using descriptive statistical methods. Comparisons between needle and cannula techniques were performed to evaluate differences in the morphology and localization of HA deposits.

Ethical considerations

The Research Ethics and Bioethics Committees issued approval (nos. CEI-10-2024 and CEB-1-2024). The study was conducted in accordance with the ethical principles of the Declaration of Helsinki. Verbal informed consent was obtained from all patients before the ultrasonographic evaluation and the aesthetic procedure. Patient data were anonymized to ensure confidentiality and stored in a secure system with restricted access. Detailed information about data handling and consent practices was provided, ensuring the ethical integrity of the study.

## Results

The cohort consisted of 27 patients, of whom 96% were women and 4% were men, with a mean age of 41 years. The doses of HA applied ranged from 0.4 to 1 cc.

Two techniques were used for HA application: the cannula technique in 11 patients (41% of the cohort) and the needle technique in 16 patients (59% of the cohort).

Several parameters were evaluated before the application of HA. A single patient could present with multiple clinical and ultrasound findings, including various concerns related to their lips, different locations of HA deposits, and multiple deposit shapes, all occurring simultaneously in the same patient.

First, findings from the physical examination revealed that 43% of the cases presented lip atrophy, predominantly affecting both lips (76%). Lip asymmetry was observed in 23% of the patients, all involving both lips. A total of 20% of patients reported a feeling of low volume and a desire for more volume; among these, 88% felt this affected both lips. Barcode lines on the upper lip were observed in 5% of cases, while another 5% exhibited exclusive hypotrophy of the lower lip. In addition, 3% of the patients presented upper lip inversion, and another 3% showed poor projection of both lips.

**Figure 3:**
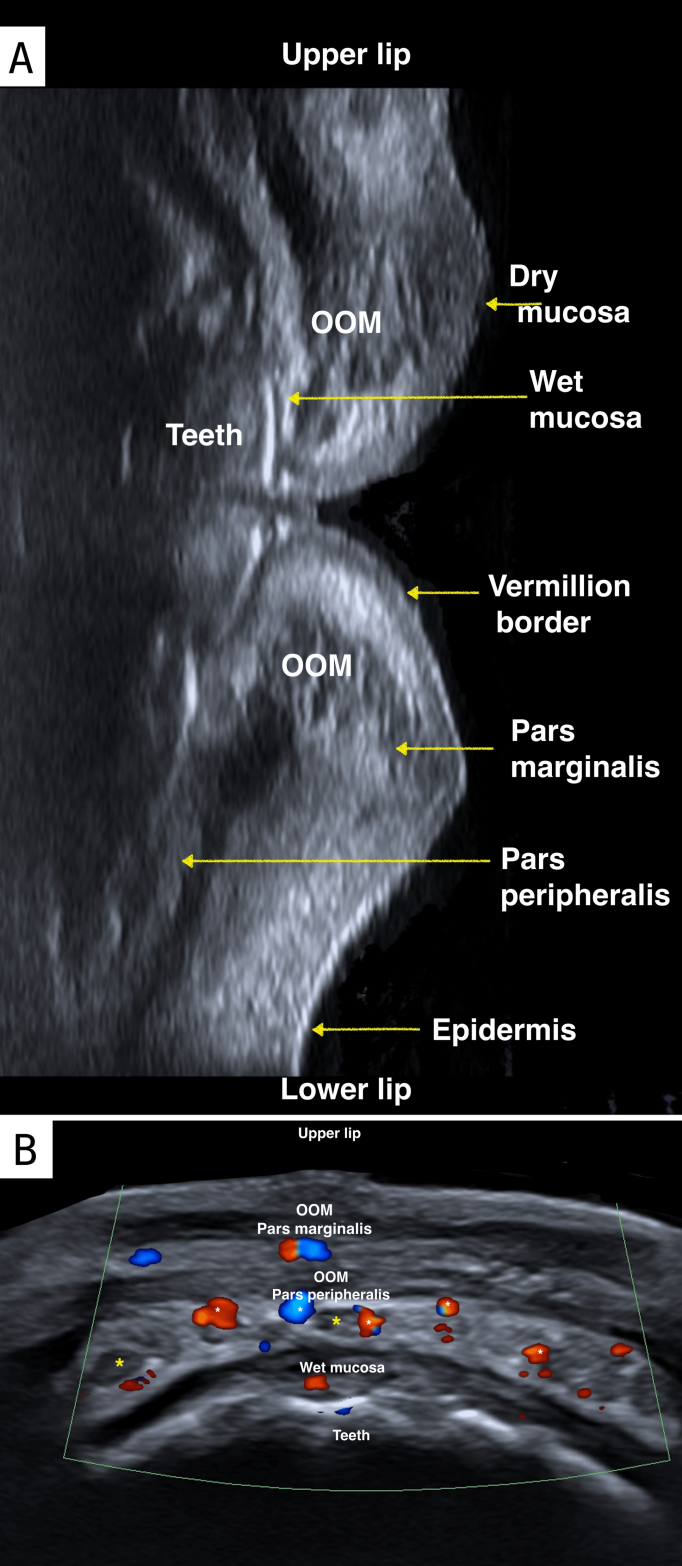
High-resolution ultrasonographic anatomy of the lips in a 21-year-old female patient A. Longitudinal high-resolution ultrasound grayscale image of the lips showcasing the detailed anatomy of the lips. B. Axial high-resolution Doppler ultrasound (Duplex Color) image of the upper lip displaying the detailed anatomy. White asterisk (*) denotes the inferior labial artery in its normal moist intramucosal course. Yellow asterisk (*) indicates minor salivary glands. OOM: orbicularis oris muscle

**Figure 4:**
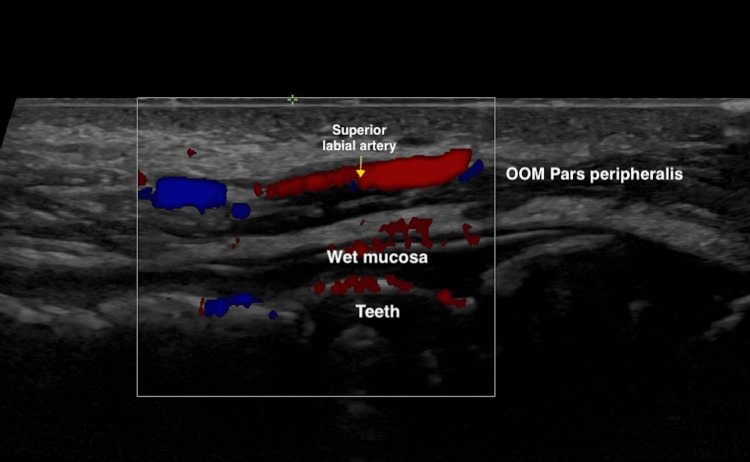
Doppler ultrasonographic identification of an aberrant superior labial artery in a 27-year-old female patient Axial Doppler ultrasound (Duplex color) image demonstrating an unusual course of the superior labial artery located within the muscular plane. OOM: orbicularis oris muscle

Second, an ultrasonographic study examined the detailed anatomy of the lips (Figure 3), including the location of the superior and inferior labial arteries (Figure 4). The findings revealed that in 81% of cases, the arteries were situated in the wet submucosa, in 15% in the superficial dry submucosa, and in 4% within the muscle.

**Figure 5:**
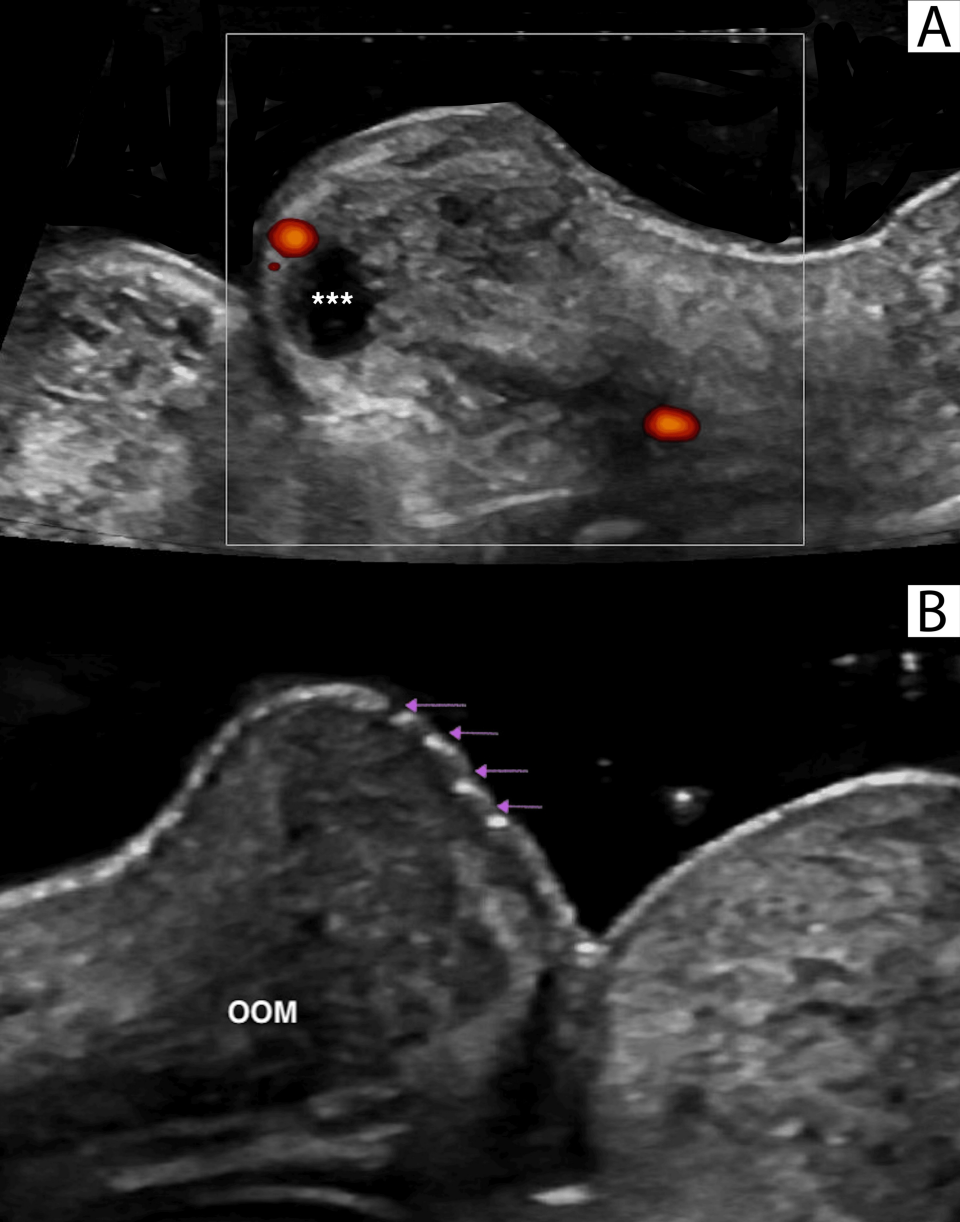
Pre-procedure ultrasonographic assessment for optimal lip volumization in two female patients aged 42 and 52 A. Longitudinal grayscale ultrasound image of the lower lip obtained before new volumization in a 42-year-old female patient. It highlights a large, visible, and palpable hyaluronic acid deposit (***) from a previous procedure that resulted in an unsatisfactory cosmetic outcome. This deposit was dissolved under ultrasound guidance before proceeding with a new volumization to achieve an optimal result. B. Longitudinal grayscale ultrasound image prior to volumization in a 52-year-old female patient. Pink arrows indicate discontinuities in the dry mucosal line, corresponding to areas of volume loss, contributing to the aged appearance of the dry mucosa in the upper lip.

Moreover, the pre-procedure grayscale ultrasonographic evaluation revealed that three patients had previously received HA fillers, which appeared on ultrasound as rounded deposits; one case also displayed an elongated appearance. Figure 5A illustrates a patient with prior filler deposits. The evaluation further identified that 48% of patients exhibited lip atrophy (Figure 5B), 26% showed poor lip projection, 15% presented with specific atrophy of the lower lip, 7% had specific atrophy of the upper lip, and another 7% displayed contour depressions. In addition, 7% of the patients showed upper lip inversion.

Other findings included low lip volume, asymmetry due to previous HA deposits (located in both wet and dry submucosa), the presence of barcode lines on dynamic exploration, HA deposits without altering lip symmetry, and visualization of hair follicles, each identified in 4% of patients. Finally, 11% of patients had normal lip structure upon ultrasonographic inspection.

**Figure 6:**
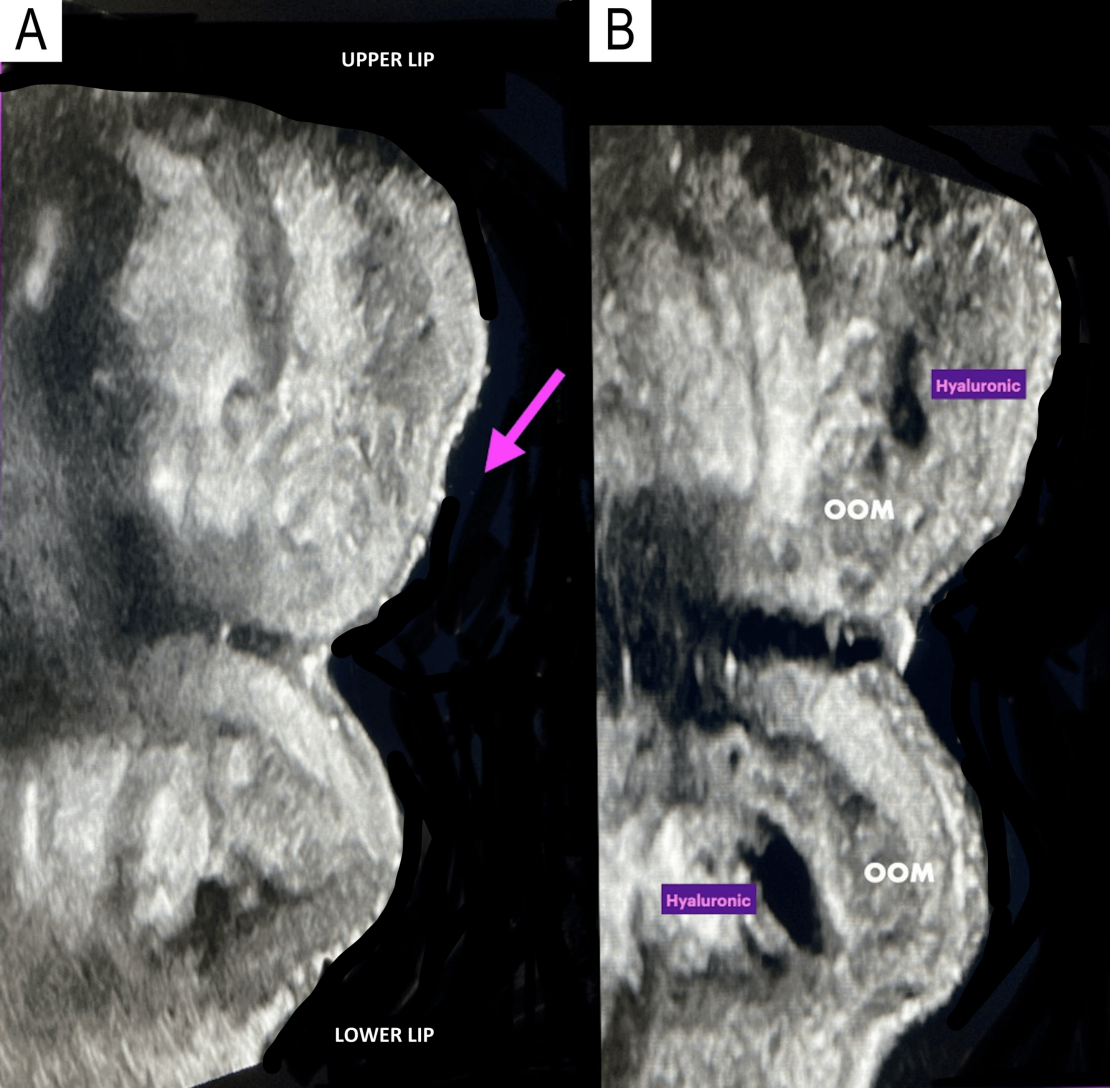
Ultrasound imaging of hyaluronic acid deposits using the needle technique in a 52-year-old female patient A. Longitudinal grayscale ultrasound image of the upper lip obtained prior to injection. The purple arrow highlights an area of volume loss, clinically presenting as a depression. B. Post-injection ultrasound image showing rounded deposits of hyaluronic acid in the upper and lower lips, located between the peripheral and marginal portions of the orbicularis muscle. The upper lip volume is restored, and the depression observed in the pre-procedure study is resolved. The lips demonstrate improved symmetry after volumization.

**Figure 7:**
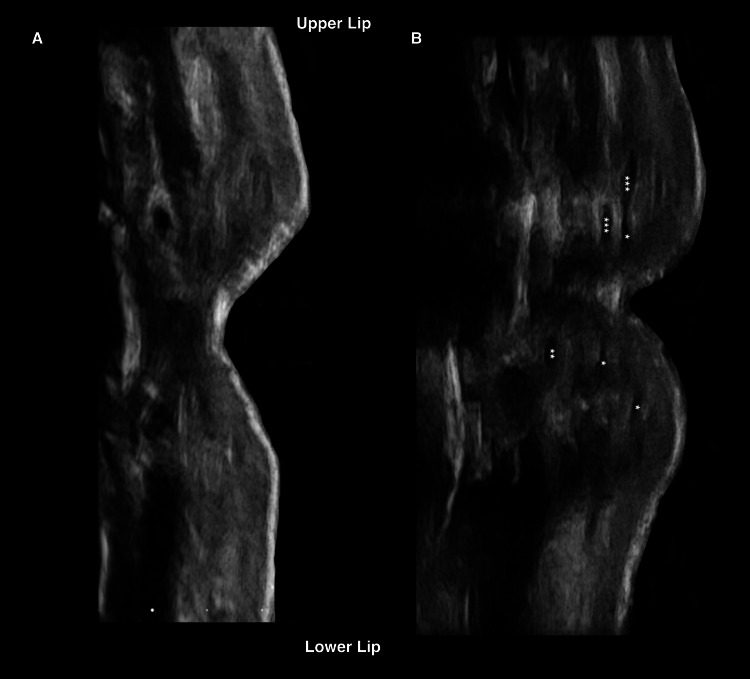
Ultrasound imaging of hyaluronic acid deposits using the cannula technique in a 36-year-old female patient A. Longitudinal grayscale ultrasound image prior to injection. The lips appear thin, and the patient desires volumization. B. Post-injection ultrasound image using the cannula technique. It shows thin, linear, hypoechoic, elongated deposits in the upper and lower lips, located along the orbicularis muscle. The resulting volumization is natural and symmetrical.

A follow-up ultrasonographic study was conducted following the injection of HA using the described techniques. Figures 6-7 illustrate the outcomes achieved with each technique.

First, the location of HA deposits was evaluated: 55% were found in the dry submucosa, 23% in the intramuscular layers, 19% in the wet submucosa, and 2% in the fat plate.

**Figure 8:**
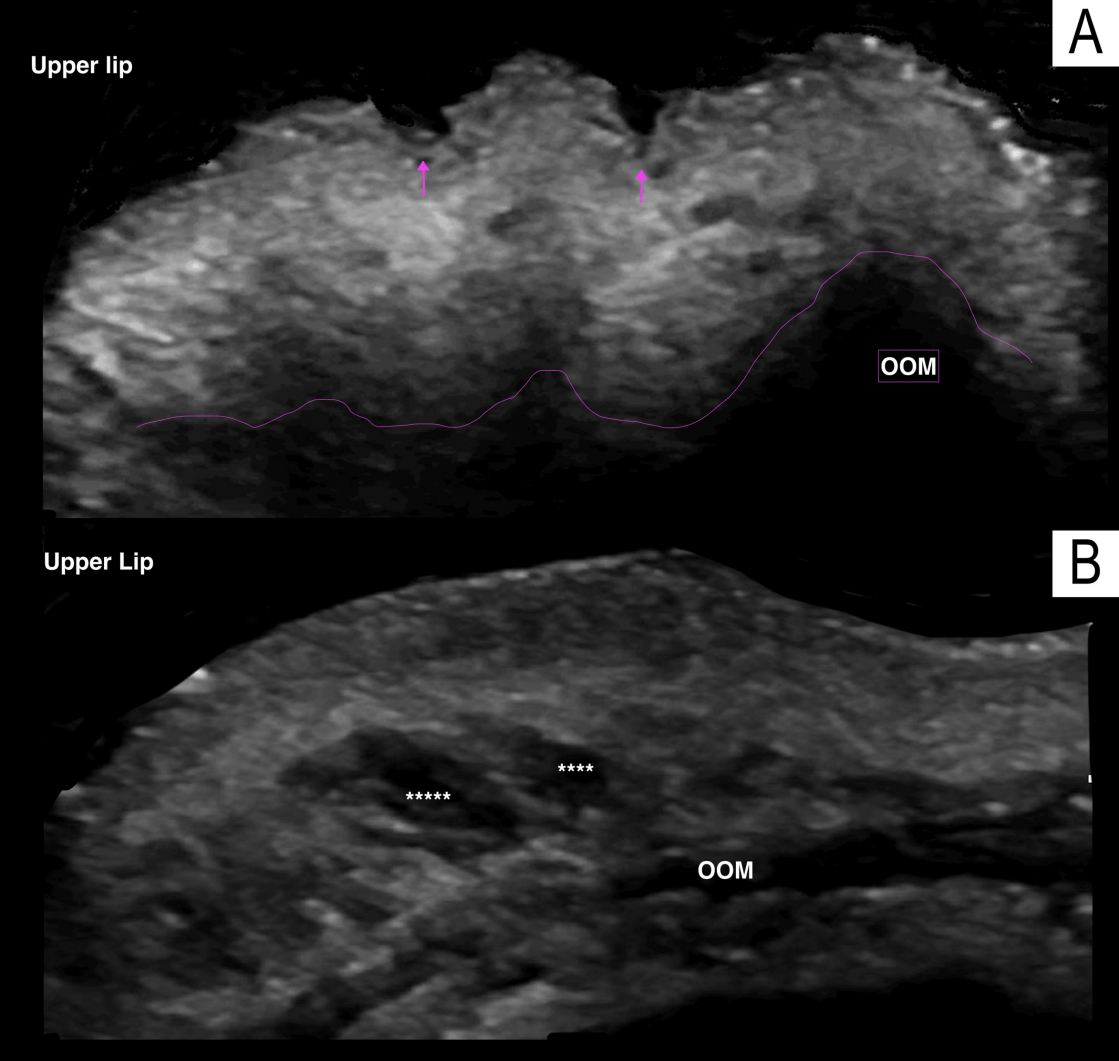
Dynamic ultrasonographic evaluation of the myomodulatory effect of fillers on the orbicularis muscle in a 66-year-old female patient Axial grayscale ultrasound image of the upper lip. A. Illustrates the dynamic exploration where asymmetric contraction of the orbicularis oris muscle generates the characteristic wrinkles known as "barcode lines." The pink line outlines the contraction of the OOM (orbicularis oris muscle), and the pink arrows indicate the barcode lines. B. Demonstrates the myomodulatory effect of hyaluronic acid deposits (****) on the orbicularis oris muscle, preventing the formation of barcode lines during contraction.

Second, the shape of the deposits was analyzed based on the technique used. A single patient may present with more than one type of HA deposit. Among the 27 patients, 25 had a single type of deposit, while two patients presented with two different types simultaneously. Elongated deposits were found in 92% (11 patients) of cases using a cannula, compared to 8% (one patient) using a needle. Conversely, round deposits were observed in 12% (two patients) of cases with a cannula and 88% (15 patients) with a needle. The two specific cases with round deposits involving the use of a cannula were patients with barcode lines, treated by placing the material above the muscle to achieve a myomodulatory effect, as demonstrated in Figure 8.

Third, all patients who presented with lip asymmetry achieved restored symmetry following the procedure. Similarly, 100% of the patients with barcode lines exhibited correction, and all cases of lip hypotrophy showed improvement, as assessed by post-procedure clinical evaluation.

**Figure 9:**
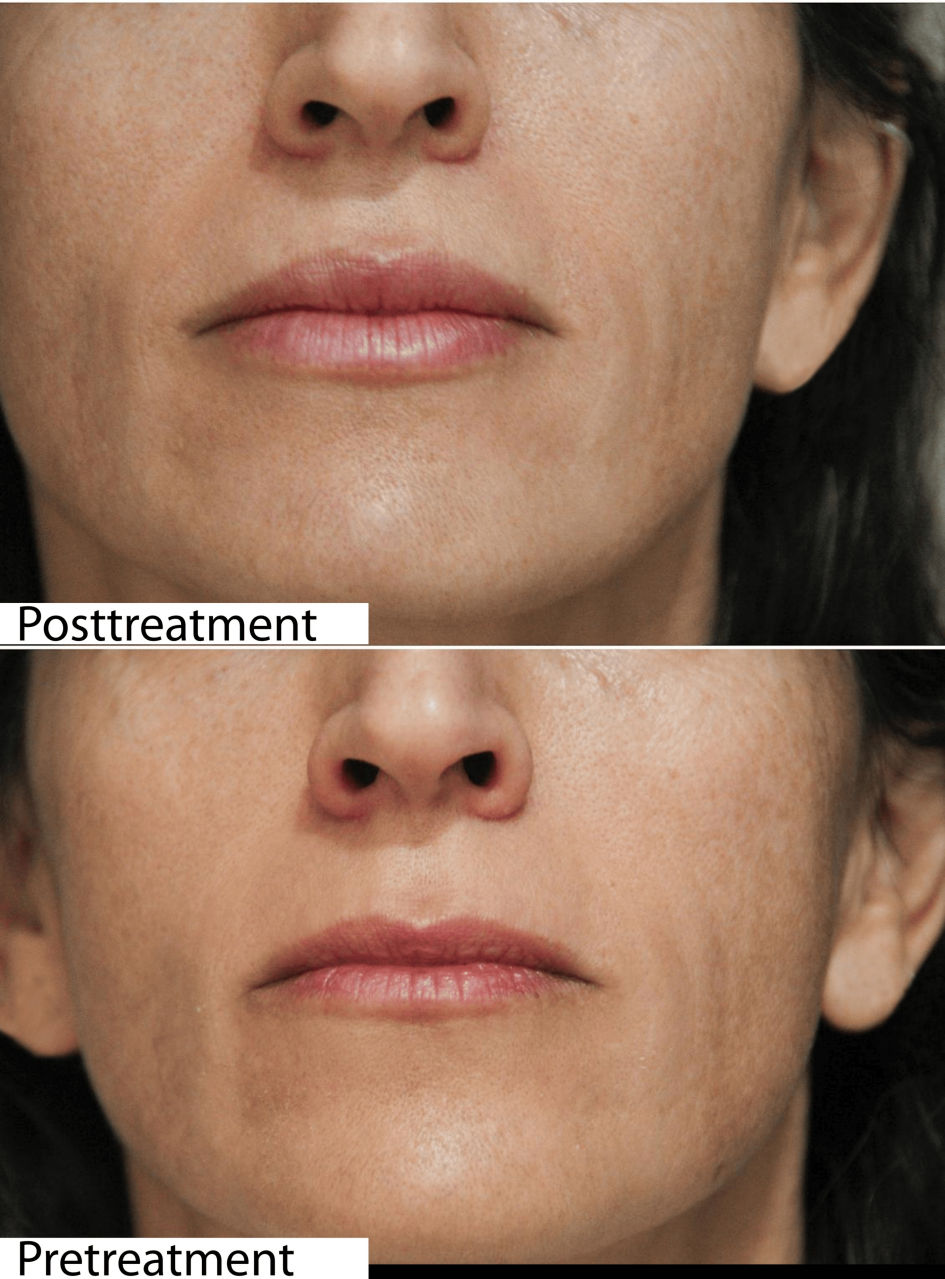
Clinical outcomes of aesthetic lip volumization using the cannula technique in a 39-year-old female patient The upper image shows the cosmetic result achieved through volumization with a cannula. Patient satisfaction was 100%.

**Figure 10:**
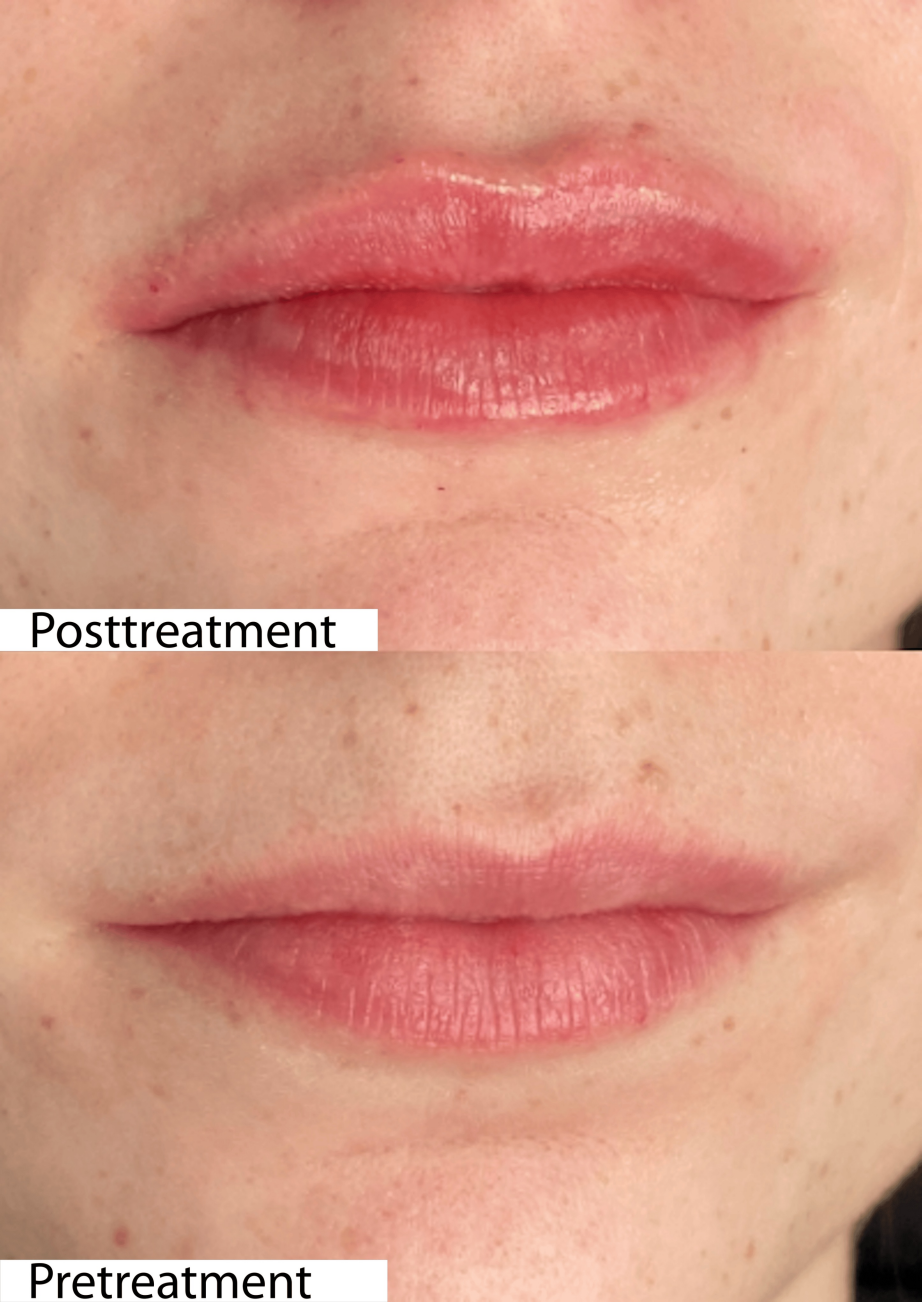
Clinical outcomes of aesthetic lip volumization using the needle technique in a 27-year-old female patient The upper image shows the cosmetic result achieved through volumization with a needle. Patient satisfaction was 100%.

Finally, a survey was conducted to evaluate patient satisfaction after the procedure. We developed a custom satisfaction scale specifically for this study. The scale ranged from 1 to 5, where 1 indicated "total dissatisfaction," 2 "dissatisfaction," 3 "moderate satisfaction," 4 "satisfaction," and 5 "complete satisfaction." The results revealed that 100% of the patients reported being satisfied with the procedure, irrespective of the technique used. Among them, 74% indicated complete satisfaction by assigning a score of 5. Figures 9-10 showcase the visible results achieved with each technique.

## Discussion

Lip volumization is one of the most sought-after aesthetic procedures today due to the importance of lips in the modern concept of beauty. This procedure aims to restore lost volume, fill perioral rhytides, and improve skin quality by increasing luminosity and elasticity [1]. In our cohort, we observed that most patients sought to restore lip volume, either due to existing atrophy or a personal desire for greater fullness. In addition, we identified a significant proportion of patients with lip asymmetry, a complaint successfully addressed through intervention. Other common aesthetic desires included eliminating rhytides on the upper lip, correcting lip hypotrophy, addressing lip inversion, and improving lip projection.

Depending on the plane in which the product is placed, different results can be achieved: the subcutaneous plane is used for hydration or restoration, the muscular plane for significant volume enhancement, and the submucosal plane for projection. In addition, injections can target the red line for lip eversion or the white line for contouring. HA injections are highly effective in increasing lip volume for at least six months, with a gradual reduction in effect by 12 months. [2]

The ideal HA for lip volumization should have specific rheological and physicochemical properties to achieve greater harmony and natural results. Regarding HA's rheological properties, elasticity should be low to medium, allowing the filler to maintain its shape while being malleable enough to integrate into the tissue and move naturally. Second, fillers should have balanced viscosity to ensure adequate gel flow and uniform distribution. Third, cohesivity, representing the adhesive forces that bind HA particles together, should be low to medium. Low cohesivity facilitates better distribution post-injection while maintaining the desired shape without dispersion. However, cohesivity can be adjusted to enhance projection and lip volumization when necessary. Finally, the product's tangent delta, representing the ratio between viscous and elastic components, should ideally be close to 1 for lips, ensuring that the gel feels natural and integrates well with the tissue [12,13].

On the other hand, the physicochemical properties of the product are equally crucial. First, the HA concentration, both insoluble and soluble, should be moderate to achieve volume without excessive rigidity. Second, the product's hydration capacity, represented by the swelling factor, should be low in the lip area, as excessive water retention can result in an edematous and unnatural appearance. Lastly, the degree of HA cross-linking determines the product's rigidity and durability. A moderate cross-linking degree ensures the gel remains in place while feeling soft to the touch [12,13].

Currently, the most common products for lip volumization include Juvederm Ultra XC, Belotero Intense, Teosyal Kiss, and Revanesse Lips. However, this study used Restylane Kysse due to its optimal characteristics for mobile areas like the lips. Restylane Kysse has been extensively studied and is noted for its appropriate rheological properties for dynamic tissues. It has an elasticity of 156 Pa, lower than standard Restylane, facilitating better integration into highly mobile tissues. Unlike other gels, Restylane Kysse has high cohesivity, allowing it to maintain the desired structure and volume without migration. A notable feature of this product is its ability to provide progressive lip volume enhancement, reaching its peak expression 30 days post-injection. Another study revealed that patients treated with Restylane Kysse reported high satisfaction and durable results, remaining effective for up to 48 weeks [14,15].

To maximize the product's benefits and adapt to individual patient needs, two HA application techniques are employed: the needle technique and the cannula technique.

Currently, both techniques are widely recognized and offer different advantages and limitations according to the literature. The cannula technique is preferred for its ability to reduce vascular complications by minimizing the likelihood of intra-arterial injection, thereby decreasing the risk of severe events such as tissue necrosis. In addition, it is associated with less pain, fewer post-injection side effects such as bruising, and greater control over HA distribution, ensuring uniform product release in the desired plane for precise volumization. However, its limitations include a learning curve for proper placement and potential challenges in achieving highly detailed lip contouring [3,4].

Conversely, the needle technique provides greater precision in detailed areas, allowing for well-defined and symmetrical lip contouring. It has also been associated with reduced product migration, which is particularly beneficial for procedures requiring fine aesthetic corrections. Nevertheless, its limitations include an increased risk of vascular complications if not performed with caution, as well as a higher likelihood of post-procedural bruising and discomfort. In addition, superficial injections with a needle require meticulous control to avoid uneven distribution of the filler [3,4].

In our study, we observed that, regardless of the technique used, 100% of cases achieved satisfaction. This suggests that both techniques are appropriate, and the choice depends on the injector’s experience and training.

Another important aspect of injection is high-resolution ultrasound, which has become a fundamental tool in aesthetic medicine for detailed facial assessment due to its ability to accurately identify different skin layers and anatomical structures such as muscles, salivary glands, and cartilage [5,6,16].

In the case of lips, ultrasonographic evaluation allows clear distinction of the peripheral and marginal portions of the orbicularis muscle, minor salivary glands, as well as dry and wet submucosal portions [7].

It also facilitates precise anatomical localization of labial arteries and allows real-time Doppler analysis to verify the course and caliber of vascular structures. This capability is crucial for preventing vascular adverse effects from HA injections. Vascular mapping with Doppler ultrasound, performed before injection, enables the identification of critical arteries, such as the superior and inferior labial arteries, minimizing the risk of intravascular injections. Similarly, superficial injections at the vermilion border, at a depth of less than 3 mm, have been shown to avoid labial arteries, enhancing procedural safety [7,11]. In our ultrasonographic study, the labial arteries were located in the wet submucosa in 82% of cases, in the superficial dry submucosa in 15%, and intramuscularly in 4%. This distribution aligns with findings from other authors, who reported submucosal distribution in 78.1%, intramuscular in 17.5%, and superficial in 2.1% [17].

Pre-procedure ultrasound serves as a valuable tool for optimizing injection planning by allowing the identification of pre-existing fillers and structural lip characteristics. In our study, ultrasound detected previous HA fillers in three patients. In addition, ultrasound objectively quantified anatomical variations such as lip atrophy, poor lip projection, and specific upper or lower lip atrophy. These findings reinforce the utility of pre-procedure imaging in tailoring treatment approaches to each patient’s needs, ensuring precise product placement and reducing potential complications.

Ultrasound is not only useful for planning HA applications but also for post-procedure monitoring. It enables precise localization of fillers, evaluation of their distribution and extent, and early detection of complications. HA deposits initially appear as anechoic areas, which may be well-defined or have irregular, heterogeneous borders, typically forming oval cavities [18-20]. Over time, these deposits become more hypoechoic and homogeneous, indicating progressive integration of HA into surrounding tissue, generally observed from week 24 post-injection. In addition, ultrasound has documented orbicularis muscle thickening after injection, contributing to lip volume enhancement [21,22].

In our study, ultrasound allowed the identification of HA deposit characteristics based on the techniques used. We believe that the variation in the ultrasonographic appearance of HA observed in our patients with each technique is associated with HA distribution and molding, described in cases of filler migration. This phenomenon occurs when the product is distributed longitudinally along the cannula to the plane of least resistance due to variations in injection pressure [23,24]. This knowledge is valuable for treating physicians, enabling them to optimize techniques and achieve more satisfactory cosmetic outcomes.

One of the most important advantages of ultrasound in aesthetic medicine is its ability to perform dynamic studies and objectively evaluate the known myomodulatory effect of fillers [25]. Two of our patients presented barcode lines due to asymmetric contraction of the orbicularis muscle. During dynamic ultrasonographic evaluation, this myomodulatory effect on the orbicularis muscle was objectively verified.

In addition, ultrasound is a valuable tool for identifying and managing complications early. Most complications described in the literature from HA lip injections are mild or moderate. These include injection site tenderness, edema, bruising, and nodule formation. Ultrasound facilitates early detection of these complications, enabling rapid and precise intervention to improve treatment safety and efficacy [2,7,20].

Finally, aesthetic lip volumization procedures, whether performed using a cannula or needle, result in high patient satisfaction. In our cohort, 100% of patients reported being satisfied with the results. Similarly, the literature reports high satisfaction rates, approaching 90% [8].

Limitations

This study has several limitations. The sample size was relatively small, with only 27 patients, which may limit the generalizability of the findings. In addition, the follow-up period was not long enough to evaluate the long-term effects and durability of HA applications. Variability in injector expertise and patient anatomy may have influenced the results, despite efforts to standardize techniques. Furthermore, the precision of ultrasound evaluation is highly dependent on the experience and skill of the operator, which could affect the accuracy of anatomical assessments and the reproducibility of findings. Future studies with larger cohorts, extended follow-up periods, and standardized protocols are needed to confirm these findings and further refine the role of ultrasound in aesthetic procedures.

## Conclusions

This study highlights how ultrasonographic exploration demonstrated that injection techniques, using either a needle or a cannula, can influence the shape and location of HA deposits. Our observations showed that elongated deposits were more commonly associated with cannula use, while round deposits predominantly resulted from needle injections. While these findings suggest a pattern, further studies with statistical analysis are needed to confirm the significance of these associations.

In addition, the procedure effectively corrected common aesthetic concerns, such as asymmetry and lip hypotrophy, restoring symmetry and improving appearance in all reported cases. Ultrasonographic evaluation before and after the procedure not only enabled precise localization of labial arteries but also identified specific characteristics of the filler deposits, significantly enhancing procedural safety and optimization.

Furthermore, this study underscores the value of ultrasound as a fundamental tool not only for personalized planning of aesthetic procedures but also for detailed follow-up and prevention of potential complications. These findings highlight the importance of carefully selecting the injection technique and conducting a thorough evaluation to achieve safe and satisfactory aesthetic results. Future studies with larger sample sizes and long-term follow-up will be essential to validate and expand upon these observations, solidifying the role of ultrasound in aesthetic medicine.
